# Supplementary material for: ARKS: chromosome-scale scaffolding of human genome drafts with linked read kmers
Source: BMC Bioinformatics. 2018 Jun 20;19:234. doi: 10.1186/s12859-018-2243-x (PMC6011487; doi:10.1186/s12859-018-2243-x)
Supplement: Supplementary file 1 — Table S1. Genomic data sources. Table S2. Summary of 10× Genomics Chromium datasets used for assemblies and scaffolding. Table S3. Summary of draft assemblies used for scaffolding with linked reads. Table S4. Contiguity and Quast summary of scaffolding a C. elegans Supernova assembly. Table S5. Contiguity and accuracy of scaffolding a Supernova assembly of the NA12878 individual. Table S6. Reconstruction of the human chromosomes in a baseline and ARKS-scaffolded NA12878 Supernova assembly. Table S7. Baseline ABySS NA24143 contig assembly metrics. Table S8. Contiguity and benchmarking analysis of scaffolding ABySS NA24143 contigs with ARKS. Table S9. Wall clock time and peak memory usage for scaffolding the Supernova C. elegans base assembly with ARKS, ARCS, fragScaff and Architect. Table S10. Wall clock time and peak memory usage for scaffolding the Supernova NA12878 draft assembly with ARKS, ARCS, fragScaff and Architect. Table S11. Wall clock time and peak memory usage for scaffolding the NA24143 Falcon+HiRise draft assembly with ARKS. Table S12. Assembly contiguity and breakpoint analysis of ARKS scaffolding of a Pacbio Falcon assembly scaffolded with Hi-C/HiRise. Figure S1. Gap size estimation in ARKS. Figure S2. ARKS gap distance estimation analysis. (PDF 446 kb) [file 12859_2018_2243_MOESM1_ESM.pdf]

Additional Information

Supplemental Tables

Table S1. Genomic data sources.

Species	Data type	Source
H. sapiens NA24143	10xG Chromium 128/151bp (BAM)	ftp://ftp-trace.ncbi.nlm.nih.gov/giab/ftp/data/AshkenazimTrio/HG004_NA24143_mother/10Xgenomics_ChromiumGenome/
H. sapiens NA24143	Pacbio Falcon assembly	ftp://ftp-trace.ncbi.nlm.nih.gov/giab/ftp/data/AshkenazimTrio/analysis/MtSinai_PacBio_Assembly_falcon_03282016/hg004_p_and_a_ctg.fa
H. sapiens NA24143	Pacbio Falcon + HiRise assembly	ftp://ftp-trace.ncbi.nlm.nih.gov/giab/ftp/data/AshkenazimTrio/analysis/Dovetail_HiRiseScaffolding_10142016/HG004_hu8E87A9_mother/falcon/hu8E87A9_mother_17Sep2016_z2vEJ/
H. sapiens NA12878	10xG Chromium 2x151bp (raw)	https://support.10xgenomics.com/de-novo-assembly/datasets/msNA12878/
C. elegans Briston N2 Strain	Reference genome	https://www.ebi.ac.uk/ena/data/view/DRX007632

Table S2. Summary of 10x Genomics Chromium datasets used for assemblies and scaffolding.

Dataset	Individual/Species	Processing step	Read pairs	Read length (bp)	Fold coverage
1	*C. elegans*	Post LongRanger	17,000,467	128/151	50
2	NA24143	Raw reads sequenced	523,746,206	151	51.2
3	NA24143	Reads from BAM	420,496,741	128/151	34.9
4	NA24143	Filtered from BAM	305,846,648	128/151	25.3
5	NA12878	Raw reads sequenced	1,598,106,419	151	156.3
6	NA12878	Post Long Ranger	1,514,291,941	128/151	136.8

Table S3. Summary of draft assemblies used for scaffolding with linked reads. The total number of scaffolds is denoted by 'n', and the number of scaffolds 500 bp and longer is denoted by 'n:500'.

Dataset	n	n:500	Largest scaffold (Mbp)	NG50 (Mbp)	Misassemblies
C. elegans Supernova	9505	2397	1.02	0.27	175
NA12878 Supernova	21774	21774	57.01	14.74	1316
Pacbio Falcon	16487	16385	22.58	4.56	3100
Pacbio Falcon + HiRise	15576	15474	65.72	14.53	3477

Table S4. Contiguity and Quast summary of scaffolding a *C. elegans* Supernova assembly. Scaffolding of the draft assembly was performed with ARCS (-*s* 98 -*c* 8 -*z* 500 -*m* 8-10000 -*e* 30000), ARKS (-*c* 8 -*j* 0.5 -*t* 8 -*z* 500 -*m* 8-10000 -*e* 30000), fragScaff (-*m* 500 -*C* 8 -*t* 8) and Architect (--*rc-abs-thr* 5, --*rc-rel-edge-thr* and --*rc-rel-prun-thr* abbreviated to "E" and "P" respectively). The most contiguous assemblies for each tool are indicated in bold. The total number of scaffolds is denoted by 'n', and the number of scaffolds 500 bp and longer is denoted by 'n:500'.

**Tool**	**Parameters**	**n**	**n:500**	**NG50 (kbp)**	**NGA50 (kbp)**	**Largest Scaffold (kbp)**	**Number of relocation misassemblies**	**Number of non-relocation misassemblies**	**Total number of misassemblies**
ARKS	k40, a0.3	9123	2015	926.03	386.81	3486.04	148	61	209
ARKS	k40, a0.5	9041	1933	1033.58	422.14	3519.47	154	64	218
ARKS	k60, a0.3	9122	2014	891.71	386.81	3485.04	148	61	209
**ARKS**	**k60, a0.5**	**9039**	**1931**	**1105.94**	**448.85**	**3519.47**	**155**	**62**	**217**
ARKS	k80, a0.3	9127	2019	891.71	386.81	3485.04	147	60	207
ARKS	k80, a0.5	9045	1937	1093.53	420.37	3519.47	153	62	215
ARCS	s98, a0.3	9124	2016	966.77	384.80	3485.04	146	61	207
**ARCS**	**s98, a0.5**	**9053**	**1945**	**1064.52**	**410.97**	**3485.04**	**147**	**60**	**207**
fragScaff	E13508, j1, u2	8798	1690	661.59	292.34	2502.12	398	77	475
fragScaff	E13508, j3, u2	8255	1147	1025.35	273.79	2504.56	771	88	859
**fragScaff**	**E13508, j6.5, u2.5**	**8098**	**990**	**1040.03**	**267.86**	**2504.56**	**842**	**117**	**959**
fragScaff	E30000, j1, u2	8928	1820	556.28	257.55	1952.43	399	73	472
fragScaff	E30000, j3, u2	8305	1197	734.06	259.48	2192.74	802	93	895
fragScaff	E30000, j6.5, u2.5	8120	1012	833.03	249.24	3065.20	892	137	1029
**Architect**	**E0.2, P0.1**	**9206**	**2098**	**410.62**	**174.28**	**1332.84**	**355**	**99**	454
Architect	E0.2, P0.2	9206	2098	410.62	174.28	1332.84	355	99	454
Architect	E0.3, P0.1	9222	2114	392.94	174.28	1332.84	343	95	438
Architect	E0.3, P0.2	9222	2114	392.94	174.28	1332.84	343	95	438

Table S5. Contiguity and accuracy of scaffolding a Supernova assembly of the NA12878 individual. Scaffolding of the baseline assembly was attempted using ARCS (-*s* 98 -*c* 5 -*m* 50-6000 -*z* 3000 -*e* 30000), ARKS (-*t* 8 -*c* 5 -*j* 0.5 -*z* 3000 -*e* 30000 -*m* 50-6000), fragScaff (-*E* 30000) and Architect (--*rc-abs-thr* 5, --*rc-rel-edge-thr* and --*rc-rel-prun-thr* abbreviated to "E" and "P" respectively). Assemblies indicated in bold are plotted in Fig. 2A, and the assembly indicated in italics is plotted in Fig. 2B and Fig. 3. The number of scaffolds 500 bp and longer is denoted by 'n:500'.

Tool	Parameters	n:500	NG50 (Mbp)	NGA50 (Mbp)	Largest scaffold (Mbp)	Number of misassemblies
ARKS	k80, a0.3	21353	23.35	8.28	74.87	1363
ARKS	k80, a0.5	21183	25.56	8.47	101.33	1447
**ARKS**	**k100, a0.3**	**21360**	**25.32**	**8.28**	**74.87**	**1359**
ARKS	***k100, a0.5***	***21207***	***25.94***	***8.47***	***101.33***	***1441***
**ARCS**	**s98, a0.3**	**21366**	**20.21**	**8.10**	**86.94**	**1363**
**ARCS**	**s98, a0.5**	**21192**	**23.09**	**8.45**	**86.94**	**1473**
**fragScaff**	**j1, u2**	**20829**	**19.06**	**7.73**	**106.43**	**1791**
fragScaff	j1.75, u2.5	20321	19.48	8.05	138.11	2147
fragScaff	j3, u2.5	19231	23.37	8.06	144.37	2889
**Architect**	**E0.2, P0.1**	**21439**	**15.05**	**7.17**	**57.01**	**1452**
Architect	E0.2, P0.2	21439	15.05	7.17	57.01	1452
Architect	E0.3, P0.1	21558	14.99	7.17	57.01	1373
Architect	E0.3, P0.2	21558	14.99	7.17	57.01	1373

Table S6. Reconstruction of the human chromosomes in a baseline and ARKS-scaffolded NA12878 Supernova assembly. ARKS scaffolding of the baseline Supernova assembly was run with parameters -k100 -j0.5 -c5 -e30000 -z3000 -m50-6000 -r0.05 -a0.5. Scaftigs from scaffolds comprising 85% (NG85) of the human genome were aligned to the GRCh8 reference genome using BWA mem [16]. The number of NG85 scaffolds covering each human chromosome, as well as the proportion of chromosome bases reconstructed by the scaffolds is shown for both assemblies.

Chromosome (sorted by #ARKS scaffolds)	Chromosome sizes (bp)	Number of Supernova (baseline) scaffolds	Sum scaffolds (bp)	Proportion of chromosome bases	Number of ARKS scaffolds	Sum scaffolds (bp)	Proportion of chromosome bases
**22**	51,304,566	1	24,215,165	47.2%	1	27,282,874	53.2%
**18**	78,077,248	3	68,423,482	87.6%	1	59,423,916	76.1%
**21**	48,129,895	2	34,048,769	70.7%	2	46,767,013	97.2%
**20**	63,025,520	3	59,048,262	93.7%	2	59,155,152	93.9%
**14**	107,349,540	9	85,454,580	79.6%	3	85,617,403	79.8%
**16**	90,354,753	10	63,227,333	70.0%	4	61,095,856	67.6%
**15**	102,531,392	9	66,742,796	65.1%	4	68,637,871	66.9%
**12**	133,851,895	10	128,061,560	95.7%	4	143,935,983	>100%
**8**	146,364,022	7	137,079,992	93.7%	4	140,829,075	96.2%
**13**	115,169,878	8	91,847,141	79.7%	5	94,935,687	82.4%
**10**	135,534,747	16	127,358,990	94.0%	5	121,023,411	89.3%
**19**	59,128,983	10	47,734,830	80.7%	6	51,904,974	87.8%
**17**	81,195,210	10	59,702,612	73.5%	6	65,400,117	80.5%
**6**	171,115,067	9	163,124,836	95.3%	6	166,383,411	97.2%
**5**	180,915,260	16	162,876,761	90.0%	6	161,153,829	89.1%
**3**	198,022,430	10	193,056,108	97.5%	6	192,350,549	97.1%
**9**	141,213,431	12	105,102,562	74.4%	7	105,817,983	74.9%
**2**	243,199,373	10	223,472,329	91.9%	7	221,709,120	91.2%
**X**	155,270,560	19	138,639,674	89.3%	7	131,154,173	84.5%
**11**	135,006,516	15	122,992,987	91.1%	8	120,078,009	88.9%
**7**	159,138,663	21	136,109,115	85.5%	8	114,034,203	71.7%
**4**	191,154,276	14	181,377,511	94.9%	8	179,479,799	93.9%
**1**	249,250,621	22	206,851,096	83.0%	13	209,511,935	84.1%
**Y**	59,373,566	NA	NA	NA	NA	NA	NA
**Total**	**3,036,303,846**	**246**	**2,626,548,491**	**86.5%**	**123**	**2,627,682,343**	**86.5%**

Table S7. Baseline ABySS NA24143 contig assembly metrics. The number of scaffolds 500 bp and longer is denoted by 'n:500'.

n:500	NG50 (kbp)	N50 (kbp)	Largest scaffold (kbp)
156,178	50.35	56.71	519.08

Table S8. Contiguity and benchmarking analysis of scaffolding ABySS NA24143 contigs with ARKS. ARKS was run with parameters -*c* 5 -*k* 100 -*j* 0.5 -*z* 3000 -*m* 50-1000 -*e* 30000 -*t* 8 -*a* 0.3. The number of scaffolds 500 bp and longer is denoted by 'n:500'.

n:500	NG50 (kbp)	N50 (kbp)	Largest scaffold (bp)	Wall clock time (h)	Peak memory (GB)
144,906	81.81	103.59	1,092,413	20.33	186.63

Table S9. Wall clock time and peak memory usage for scaffolding the Supernova *C. elegans* base assembly with ARKS, ARCS, fragScaff and Architect. Benchmarking for the most contiguous assemblies are shown. ARKS, fragScaff and the BWA mem [16] alignments were run using eight threads, while ARCS and Architect are single-threaded. The peak memory step for ARCS, fragScaff and Architect was the alignments of the linked reads to the draft assembly, while the peak memory step for ARKS was the main scaffolding pipeline.

Tool	Wall clock time (min)	Peak Memory (GB)
ARKS	11.08	1.42
ARCS	40.08	1.26
fragScaff	43.02	1.26
Architect	51.42	1.26

Table S10. Wall clock time and peak memory usage for scaffolding the Supernova NA12878 draft assembly with ARKS, ARCS, fragScaff and Architect. Benchmarking for the most contiguous assemblies are shown. ARKS, fragScaff and the BWA mem [16] alignments were run using eight threads, while ARCS and Architect are single-threaded.

Tool	Wall clock time (h)	Peak Memory (GB)
ARKS	10.50	6.68
ARCS	58.90	6.42
fragScaff	71.28	24.89
Architect	96.89	34.40

Table S11. Wall clock time and peak memory usage for scaffolding the NA24143 Falcon+HiRise draft assembly with ARKS. Benchmarking for the most contiguous assembly is shown.

**Wall clock time (h)**	3.85
**Peak memory (GB)**	7.19

Table S12. Assembly contiguity and breakpoint analysis of ARKS scaffolding of a Pacbio Falcon assembly scaffolded with Hi-C/HiRise. Tigmint was run with parameters *w*=2000 *n*=2 and ARKS was run with parameters -*t* 8 -c 5 -*j* 0.5 -*z* 3000 -*e* 30000 -*m* 50-1000. Assemblies indicated in bold are plotted in Fig. 5. The number of scaffolds 500 bp and longer is denoted by 'n:500'.

**Assembly**	**ARKS Parameters**	**n:500**	**NG50 (Mbp)**	**NGA50 (Mbp)**	**Largest scaffold (Mbp)**	**Number of misassemblies**
**Falcon**	**N/A**	**16385**	**4.56**	**4.11**	**22.58**	**3100**
**Falcon + ARKS**	**k40, a0.3**	**15551**	**15.00**	**7.92**	**70.49**	**3314**
Falcon + ARKS	k60,a0.3	15680	12.99	7.57	62.81	3247
**Falcon + Tigmint + ARKS**	**k40,a0.3**	**16480**	**16.71**	**10.02**	**70.49**	**3053**
Falcon + Tigmint + ARKS	k60,a0.3	16598	15.06	9.90	67.20	3031
**Falcon + HiRise**	**N/A**	**15474**	**14.53**	**8.15**	**65.72**	**3477**
Falcon + HiRise + Tigmint	N/A	16463	13.94	8.15	59.63	3353
**Falcon + HiRise + ARKS**	**k40, a0.3**	**15118**	**23.09**	**9.26**	**104.20**	**3612**
Falcon + HiRise + ARKS	k60, a0.3	15227	23.09	9.42	104.20	3538
**Falcon + HiRise + Tigmint + ARKS**	**k40, a0.3**	**16080**	**30.01**	**10.02**	**104.20**	**3471**
Falcon + HiRise + Tigmint + ARKS	k60, a0.3	16193	28.46	10.02	104.20	3413

Supplemental Figures

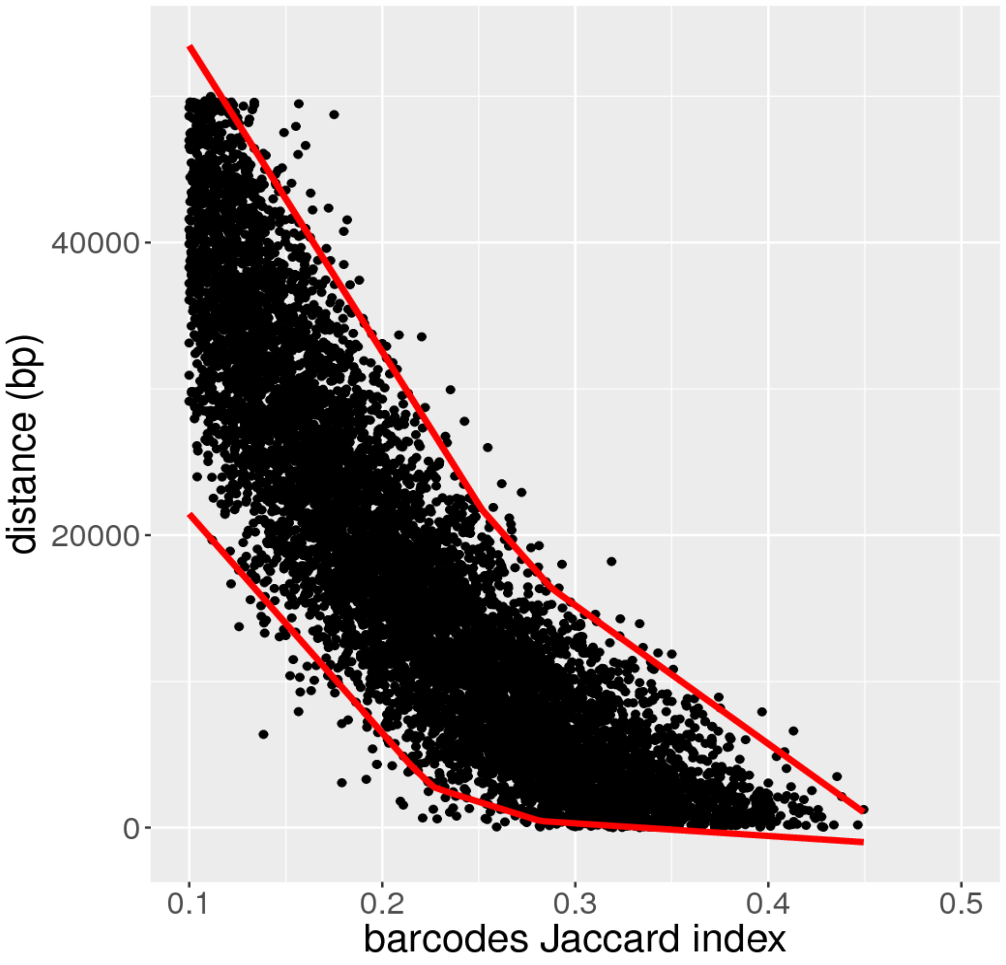

Figure S1. Gap size estimation in ARKS. To parameterize the relationship between number of shared barcodes and distance, ARKS measures the distance between head and tail regions of the same contig and records the corresponding barcode Jaccard index. Here, we show an example set of intra-contig distance/barcode samples for Chromium reads mapping to ABySS assembly of the NA24143 dataset, using ARKS parameters `-c5 -e30000 -z3000 -m50-1000`. Minimum and maximum distance bounds are estimated using the 1st and 99th percentile of the observations (red lines).

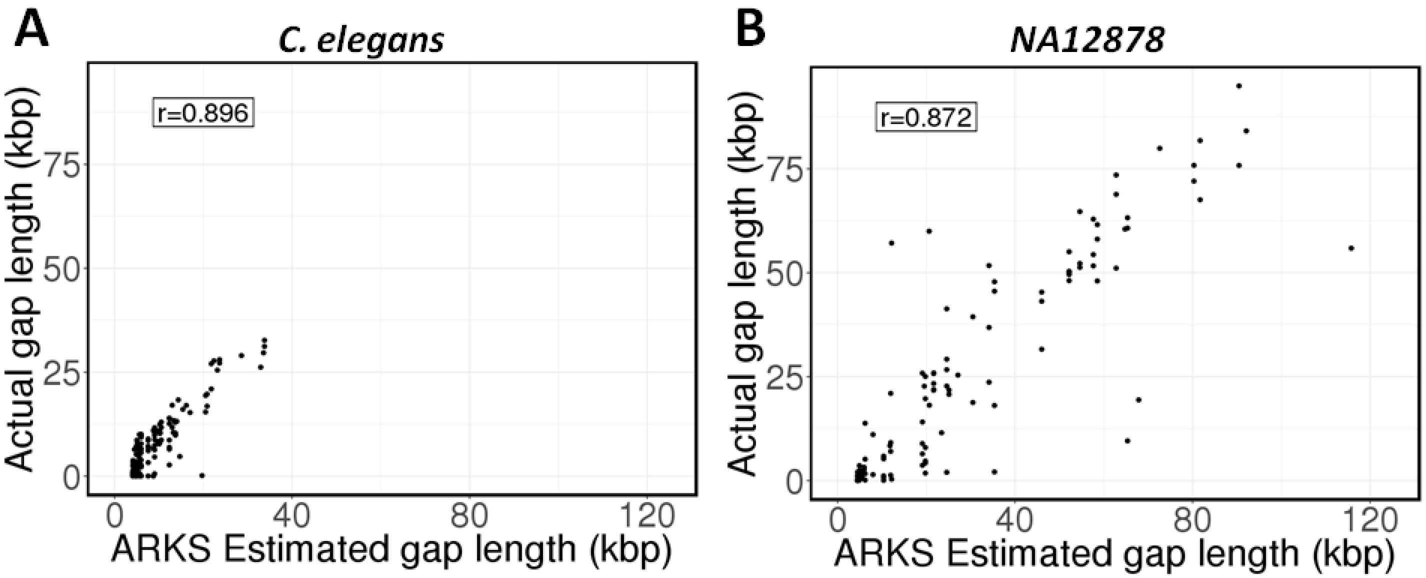

Figure S2. ARKS gap distance estimation analysis. In separate experiments, ARKS was run on the (A) *C. elegans* (*-D -c* 8 *-z* 500 *-m* 8-10000 *-e* 30000 *-k* 60 *-a* 0.5 *-j* 0.5) and (B) NA12878 (*-D -c* 5 *-e* 30000 *-z* 3000 *-m* 50-6000 *-k* 60 *-a* 0.5 *-j* 0.5) Supernova assemblies, using the gap distance estimation option. The ground truth (Actual gap length) was derived from aligning adjacent contigs within a scaffold, to their respective reference genome sequence. The Pearson correlation coefficient (r) was calculated using the ARKS-estimated and actual distances for both datasets.
